# Detection and psychoprophylaxis with beneficiaries who are in the first stages of artistic expression- children and adolescents aged 2 to 18 years, of both sexes, from different cultures, social backgrounds and education levels are included

**DOI:** 10.1192/j.eurpsy.2023.1732

**Published:** 2023-07-19

**Authors:** C. Emilia

**Affiliations:** Centru Comunitar Județean Complex Servicii Sociale Comunitare pentru Copii și Adulți Cluj, CONSILIUL JUDEȚEAN CLUJ DIRECŢIA GENERALĂ DE ASISTENŢĂ SOCIALĂ ŞI PROTECŢIA COPILULUI CLUJ, Cluj-Napoca, Romania

## Abstract

**Introduction:**

If children are taught early on to control their emotions, that can prevent problems that cause disturbing emotions: violence, suicide, drug abuse, etc. The artistic experience, just like the religious one, is an essential experience of the human being. Art ,occupational, play therapy concerns itself with the information that the images have to offer regarding their author.

**Objectives:**

Our goal is to discover new perspectives and sources of inspiration to establish new prevention and recovery methods and techniques to advance in defining the importance of resilience in personal development. The development and maintenance of social skills will thus be the necessary conditions for improving adaptability and the capacity for personal transformation, without forgetting at the same time the effectiveness of the process of resocialization and recovery of juvenile delinquents.

**Methods:**

The child is stimulated in the problem-solving and the decision-makings strategies, in order to achieve formal diversifications. In the preventive activities we include all activities involving nonverbal communication and holistic engagement of people in creative activities, specific to visual arts (plastic, decorative design and multimedia), facilitating school reintegration or optimizing the school situation, completing the general and specialized culture.

**Results:**

The individual skills are traced by the specific means of the visual arts; moreover, we care about the individual capacities, the freedom to follow his own destiny, encouraging the joy to manifest creatively on several levels of difficulty in any activity. These activities lead to changes in the attitudes towards the work or the discovery of recreational activities and the use of leisure time.

**Image 2:**

**Figure:**
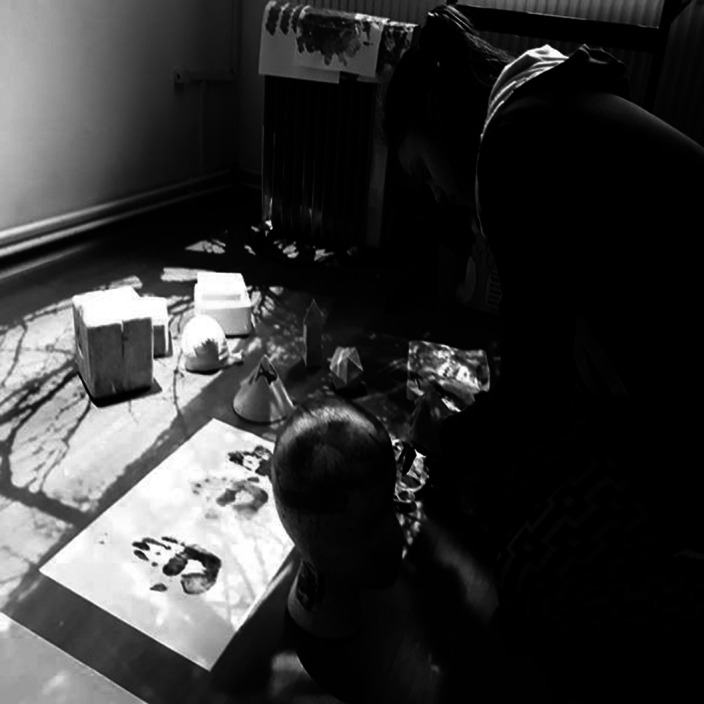

**Conclusions:**

Considering the diversity of the child`s non-verbal communication, art therapy, occupational and play therapy are not a mere accessory method within the therapeutic process of the emotional disorders of children, but a mandatory condition of it. The development and the maintenance of social abilities will thus be the necessary conditions of an improved adaptability and of the capacity of personal transformation, “Social Cohesion” is a Common Goal for Psychiatry, and art, occupational , play therapies.

**Disclosure of Interest:**

None Declared

